# Posthemorrhagic hydrocephalus in extremely low birth weight infants: Ommaya reservoir vs. ventriculoperitoneal shunt

**DOI:** 10.1007/s00381-015-2754-y

**Published:** 2015-05-28

**Authors:** Ralf-Bodo Tröbs, Volker Sander

**Affiliations:** Klinik für Kinderchirurgie, Marienhospital Herne, St. Elisabeth Gruppe Rhein-Ruhr, Ruhr-Universität Bochum, Widumer Str. 8, D-44627 Herne, Germany

**Keywords:** Posthemorrhagic hydrocephalus, Ommaya reservoir, Ventriculoperitoneal shunt

## Abstract

**Purpose:**

The aim of this study was to analyze morbidity and initial surgery in infants with posthemorrhagic hydrocephalus (PHH) by comparing infants who were treated with a subcutaneous cerebrospinal fluid reservoir (Ommaya reservoir = CSF_R) with infants who primarily received a ventriculoperitoneal shunt (VPS).

**Method:**

Inclusion criteria were infants born between January 2006 and June 2014 who had a diagnosis of intraventricular hemorrhage (IVH) and underwent surgical intervention for hydrocephalus.

**Results:**

Twenty-five infants, with a median gestational age (GA) of 26.5 (28 ± 4) weeks and a median birth weight (BW) of 980 g (1205 ± 837), were included. The median umbilical artery pH (UApH) was 7.30 (7.20 ± 0.25). The median Apgar score at 10 min was 8 (7.4 ± 2). Twenty-five peri- and postnatal adverse events were encountered preoperatively. The IVH grades were grade II (*n* = 1), grade III (*n* = 17), grade IV (*n* = 6), and unknown grade (*n* = 1). Primary treatment consisted of CSF_R (*n* = 18) or VPS (*n* = 7) placement. There was a statistically significant difference between the postnatal ages of infants with CSF_R (32.5 days; 42 ± 28) and infants with VPS (163 days; 161 ± 18). Furthermore, we found a difference regarding GA but not BW between both groups. Arrest of PHH with shunt independence occurred in two infants from the CSF_R group (11 %).

**Conclusions:**

In the present study, early insertion of CSF_R allowed stabilization of the infants and thus postponement of permanent VPS insertion. However, in a subgroup of patients, PHH develops over a more prolonged course, and VPS insertion can be performed initially without the need for CSF_R.

## Introduction

Posthemorrhagic hydrocephalus (PHH) is one of the most serious complications of premature labor and postnatal intraventricular hemorrhage (IVH) [1]. The sequelae of PHH, such as neurological deficit, epilepsy, and cognitive impairment, can be extremely common in the subgroup of patients with hydrocephalus [2].

Previous studies have shown that neither serial cerebrospinal fluid drainage nor treatment with furosemide, acetazolamide, or streptokinase-based fibrinolysis can improve infant outcomes [3]. Thus, surgical diversion of cerebrospinal fluid (CSF) is the treatment option of choice.

Surgical treatment for PHH can be performed in two steps. For the initial treatment, temporary procedures for cerebrospinal fluid (CSF) drainage can be performed. These include percutaneous lumbar or transfontanellar ventricular CSF aspiration, external drainage, or placement of a ventricular catheter with a subcutaneous CSF reservoir [CSF_R].^1^ CSF withdrawal is performed by simple needle aspiration on an as-needed basis [3]. For the definitive treatment of PHH, however, ventriculoperitoneal shunt (VPS) insertion is the preferred method for most surgeons worldwide.

The aim of this study was to characterize perinatal characteristics, perioperative management, morbidity, and mortality in infants with PHH. Patients who were primarily treated with a temporary CSF reservoir system were compared to a group of patients primarily treated with a VPS.

Data from the present study were interpreted under the background of a national survey on the management of PHH [4] and large recently published reviews and multi-institutional series [2, 3, 5].

## Materials and methods

In this retrospective study, we evaluated all infants who had been treated for PHH between January 2006 and June 2014 in our department. During the observation period, 26 infants were identified; 25 of whom were included for further analysis. One patient was excluded due to missing data. We evaluated clinical records and operative reports to extract data on perinatal course, timing of operation, and in-hospital morbidity and mortality.

Any infant born before 37 weeks (wk) gestation age (GA) was defined as *preterm*.

A birth weight (BW) below 1500 g was considered very low (*VLBW*), and a BW below 1000 g was considered extremely low (*ELBW*).

The primary outcome for the CSF_R (CSF/Ommaya reservoir) group was defined as the duration of CSF_R insertion and conversion rate to permanent VPS.

IVH causing ventricular dilatation (grade III) or ventricular dilatation with involvement of the adjacent parenchyma (grade IV) was considered severe IVH. Events influencing cardiorespiratory status that could have caused or contributed to the pathophysiology of IVH and PHH were analyzed (peri- and postnatal morbidity). Other typical serious problems, such as premature retinopathy and bronchopulmonary dysplasia, were not investigated.

CSF surgery-related mortality was defined as any death occurring during the patient’s stay in the Department of Pediatric Surgery.

### Surgery

The indication for surgery was established after the development of convincing clinical symptoms and signs of elevated intracranial pressure. Serial ultrasound (US) investigations were performed prior to surgery. The indication for surgical treatment was always established in close cooperation with the neonatologist. Surgery was performed under general anesthesia with routine administration of prophylactic antibiotics. The preferred site of ventricular catheter insertion was the most distended lateral ventricle, via frontal access. The ventricular catheter was inserted via a burr hole. Ultrasound guidance was used to navigate and to control the position of the ventricular catheter. We used either right-angled or straight CSF reservoir systems (Medtronic Inc., Goleta, CA, USA) and 3- to 5-cm pieces of shunt tubing. Careful attention was paid to the maintenance of the infant’s body temperature, and a meticulous surgical technique was applied to protect the soft tissues and skin of these vulnerable patients. For VPS placement, we used position-sensitive Christoph Miethke paediGAV valves (Aesculap, Tuttlingen, Germany) with opening pressures of 4 or 9 cm H_2_O in a horizontal position and 24 cm H_2_O in a vertical position. Prior to shunt surgery, we ruled out CSF infection by repeated negative microbiological cultures, and a CSF protein <1.5 g/L was accepted for valve insertion.

### Statistics

Data are expressed as the median or the arithmetic mean ± standard deviation with the range (minimum-maximum) noted in parentheses. Sample sizes differing from the entire group of patients are indicated.

A significant difference between the median and mean indicates a skewed distribution. The non-parametric Wilcoxon rank-sum test was used to compare samples of two unrelated groups. The two-tailed *p* value was used, and *p* values <0.05 were considered statistically significant [6].

## Results

All infants were born in a perinatal center without a pediatric surgical unit and were transferred to our hospital by local, specialized neonatal transport teams. The average case load was three cases per year. The cohort included 8 female and 17 male newborns (male predominance 2:1).

### Epidemiologic and anthropometric data (Table 1)

The present cohort included seven twins (five delivered as the first infant, and two delivered as the second infant) and two triplets (delivered as the first and the third infant).

**Table 1:** Baseline patient characteristics

Parameter	Median (mean ± SD)	Range
Gestational age (weeks)	26.5 (28 ± 4)	23–29
Birth weight (g)	980 (1205 ± 837)	470–3480
Apgar score at 10 min	8 (7.4 ± 2)	2–10
Umbilical artery pH	7.20 (7.20 ± 0.25)	6.68–7.49
Grade of IVH	3 (3.2 ± 0.5)	2–4

*IVH* intraventricular hemorrhage

All neonates were born by caesarean delivery (CS) (100 %). After a median GA of 26.5 weeks (28 ± 4; range 23–39), the median BW was 980 g (1205 ± 837; range 470–3480). Nine newborns (36 %) had a BW below 1500 g (VLBW), and 14 infants (48 %) had a BW below 1000 g (ELBW). In addition, 23 infants (92 %) were delivered preterm and 2 at term. The median umbilical artery pH (UApH) was 7.20 (7.20 ± 0.25; range 6.68–7.49; *n* = 15), and the median Apgar scores were 7 (6 ± 3; range 1–9) at 5 min and 8 (7.4 ± 2; range 2–10) at 10 min. In three infants, UApH was below the critical value of 6.99, and one newborn had a borderline UApH between 7.0 and 7.11 [7]. One patient had IVH grade II, 17 had grade III, 6 had grade IV, and 1 had an unknown grade IVH (median 3; 3.2 ± 0.5).

### Peri- and postnatal morbidity prior to initial surgery for PHH (Table 2)

At least 25 serious events, conditions, or malformations were encountered in the peri- and postnatal periods prior to insertion of the CSF shunt. Prenatal intracerebral bleeding was detected in one infant. For three infants, cardiopulmonary resuscitation (CPR) was required immediately after delivery. Two infants suffered from severe postnatal septicemia.

**Table 2:** Delivery mode, gestation number, and infant complications

Parameter	Frequency
Caesarean delivery	100 %
Multiple gestations (*n* = 9)	7 twins 2 triplets
Peri- and postnatal events (*n* = 25)	1 prenatal intracerebral bleeding 3 asphyxia/postnatal CPRs 3 abdominal sepsis/events 3 PDA closures 4 ibuprofens for PDA 3 major congenital heart defects 5 thoracic events 1 traumatic delivery 2 septicemias

*CPR* cardiopulmonary resuscitation

*PDA* patent ductus arteriosus

In seven infants, patent ductus arteriosus (PDA) was detected and treated (four with ibuprofen and three with surgical PDA ligature), and in three infants, major congenital malformations of the heart were detected (two ventricular and one atrial septal defect). Furthermore, five infants had thoracic complications (two pneumothorax, two pulmonary bleeding, and one bilateral hemothorax), and three infants had abdominal complications (necrotizing enterocolitis, focal intestinal perforation, and volvulus).

### Comparison of CSF_R vs. VPS (Fig. 1, Table 3)

Patients were divided into two groups according to the initial treatment received.

**Fig. 1:**
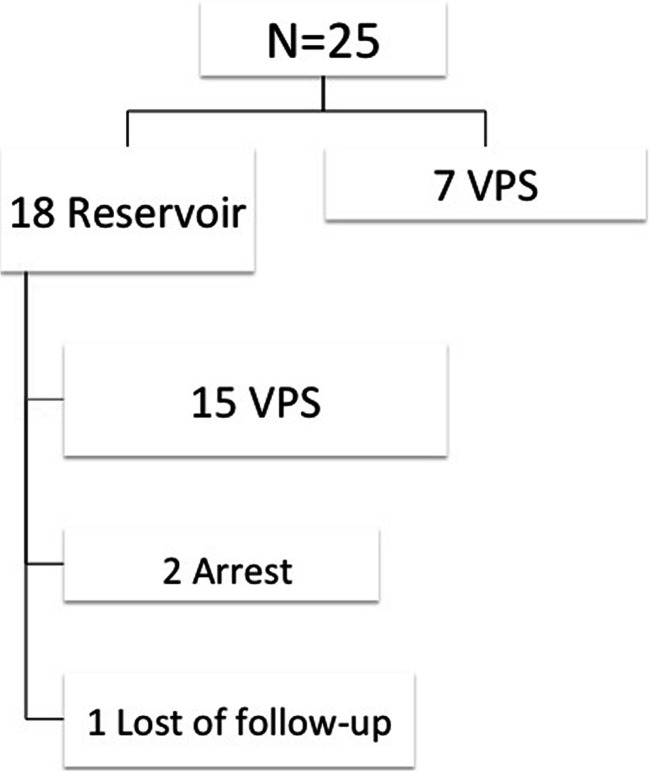
The two groups. Follow-up after reservoir insertion

**Table 3:** Comparison of baseline characteristics and sequence of CSF drainage in both groups

	Ommaya reservoir	Primary VPS	Significance
Patients	*n* = 18	*n* = 7	**–**
Gestational age (weeks)	26 (27 ± 4) (range 23–39)	29 (29 ± 5) (range 23–37)	*p* = 0.057
Birth weight (g)	980 (1202 ± 879) (range 470–3480)	1080 (1548 ± 1161) (range 675–3440)	*p* = 0.7
Median grade of IVH	III	III	**–**
Age at insertion (days)	32.5 (42 ± 28) (range 19–126)	163 (161 ± 118) (range 26–318)	*p* = 0.028

*IVH* intraventricular hemorrhage

*VPS* ventriculoperitoneal shunt

1. Temporary insertion of an Ommaya reservoir (CSF_R; *n* = 18; 72 %)
  In 18 infants, temporary insertion of a ventricular catheter connected to a subcutaneous CSF_R was performed at the age of 32.5 days (42 ± 28; range 26–318). The CSF_R was left in situ for 55 days (57 ± 30; range 6–126).
  For 15 patients in this group, the CSF_R was explanted and replaced with a VPS. No CSF_R/CSF infections were observed; however, one infant was treated with external drainage shortly after conversion to VPS. For 1 infant in this group, dysfunction of the CSF_R led to early replacement with a VPS. In 2 of 17 (12 %) patients, hydrocephalus arrested (1 Ommaya reservoir was removed, and 1 was left in situ, ready for explantation); 1 patient with a CSF_R changed caregivers and was lost to follow-up.
2. Primary VPS insertion (*n* = 7; 28 %)
  In seven infants, placement of a CSF_R was avoided, and VPS were inserted for CSF drainage at the age of 163 days (161 ± 118; range 26–318). For five infants, the VPS remained in place for at least 14 months until the first shunt revision. Two early revisions were required 1 month after the primary insertion, one for a gram-negative shunt-associated infection and one for temporary occlusion of the peritoneal catheter.
  Baseline characteristics and timing of the surgery for the CSF_R and VPS groups are outlined in Table 3. Differences in GA between the two groups approached statistical significance (*p* = 0.057). The BW between groups was not significantly different (*p* = 0.70). However, there was a significant difference between the two groups with respect to the time of CSF_R or primary VPS insertion (*p* = 0.028).

### Mortality

No perioperative or surgery-related mortality occurred.

### Limitations

This institutional series is limited by the retrospective character of the study and small sample sizes. Surgery was performed exclusively by or under the direct supervision of the authors (VS and RBT).

## Discussion

IVH remains a serious problem in VLBW infants, and a significant proportion of affected infants develop progressive and symptomatic hydrocephalus.

Clinical signs of symptomatic PHH include a rapidly enlarging orbitofrontal head circumference, increased splaying of the cranial sutures, a protruding and tense fontanelle, and worsening clinical status [5].

In cases of premature labor, it has been demonstrated that the incidence of IVH can be decreased by maternal corticosteroid administration before the 34th week of gestation [8, 9]. IVH remains a serious problem, and 25 % of affected infants develop ventricular dilatation, which can resolve spontaneously or become progressive and require further treatment [10]. It has been shown that the need for surgery depends primarily on the severity of IVH.

Generally, shunt insertion in very premature infants after ventricular hemorrhage is associated with elevated rates of shunt failure and shunt infection [3, 5].

Soon after intraventricular bleeding, blood degradation products and debris are present in considerable amounts in the CSF. Under these conditions, placement of a VPS with a valve mechanism carries an elevated risk of shunt obstruction [3]. In addition, in many of these very small infants, the permanent shunt-valve system is not well-tolerated, and skin breakdown and shunt infection may occur. Therefore, we prefer to perform initial insertion of small temporary subcutaneous CSF reservoirs, an approach that was used in 18 of 25 infants in the present study. In a large North American multicenter study, two-thirds of patients were initially treated with a CSF reservoir, and a smaller fraction underwent placement of a ventriculosubgaleal shunt or external drain [3]. Temporary placement of a CSF reservoir was the preferred initial treatment for PHH in 79 % of about three-thirds of German neurosurgery and 70 % of pediatric surgery departments. Primary placement of a VPS was preferred only by a small minority of responders [4].

Recently, neuroendoscopic lavage and third ventriculostomy have been evaluated for the treatment of PHH [10, 11].

In patients with PHH, VPS placement is associated with an elevated risk of complications (including CSF leakage, infection, obstruction, and skin breakdown). In our series, only one case of mechanical dysfunction and one case of external drainage for infection after conversion to VPS were documented. This is lower than reported in a recently published review, in which the median rate of shunt infection per surgery was found to be approximately 8 % [2].

However, in a previous series, only 1 of 20 (5 %) VLBW infants developed CSF infection after a total of 71 tapping procedures per patient [12].

The low rate of mechanical complications in the present study may be explained by the retrospective nature of this investigation. Small subcutaneous CSF collections without compromise of the reservoir function were not of major clinical importance and may have remained undetected. According to the literature, reservoir complication rates may be as high as 22 % [3].

In our study, 15 of 17 (88 %) patients underwent conversion to a permanent shunt (including 1 patient who was lost to follow-up). In 2 of our patients, symptomatic hydrocephalus resolved, and no attempt at VPS insertion was made. Our conversion rate is comparable to the result of another single institution series, which had an 85 % rate of conversion to a permanent shunt [12]. However, our conversion rate to permanent shunt was higher than that reported in a recent large multicenter study, in which conversion to VPS occurred in 69 % of infants [3]. These differences can be explained by patient selection and criteria for treatment, which vary by surgeon and institution. Thus, other institutional series have reported conversion to a permanent shunt in 100 % of infants [3]. In a meta-analysis, shunt independence was achieved in 3.2 to 9 % of infants [2].

The vast majority of infants in our series had very or extremely low birth weights. Very immature newborns are predisposed to a high rate of comorbidities and clinical problems. In some of our patients, UApH was below 7.11. This is consistent with the results of a recent meta-analysis, in which a low UApH was associated with an increased risk of cerebral morbidity (i.e., IVH and periventricular leukomalacia). A clear dose-response relation between pH and morbidity is assumed, with the strongest association at a threshold of pH 7.00 [13].

Severe neonatal septicemia, one of the major causes of IVH, was found in two patients in our cohort [8]. In our investigation, we found a high rate of thoracic and abdominal events/infection, and PDA treatment was required in a considerable number of patients. According to the literature, prophylactic intravenous indomethacin, which is used to treat many ELBW infants, does not affect the risk of IVH. However, persistent fetal circulation contributes to hypercapnia and acidosis, with an increased risk of IVH and subsequent hydrocephalus [5].

All patients in our series were delivered by caesarean section (CS), and more than one-third of the infants were twins or triplets. CS is thought to decrease the risk of IVH [5]. The majority of affected multiple infants in our series were the first-born neonates. The hypothesis that second-born twins are at a disadvantage cannot be confirmed by the literature [14]. According to recently published studies, the mode of delivery and multiple gestations were not associated with an elevated risk of severe IVH [8, 14]. Furthermore, the outcome of the first presenting twin seems to be similar to that of the second delivered twin. Univariate analysis has revealed that early sepsis and the occurrence of pneumothorax are the main causes of IVH in ELBW infants [8]. Other maternal factors (e.g., smoking or socioeconomic status) have not been found to be associated with a higher rate of IVH [8].

Our series included 25 infants over 8.5 years, yielding an average annual frequency of three cases. According to a recently published survey, neurosurgery and pediatric surgery units reported a case load between 1 and 20 cases per year (median 7 to 9) [4]. Furthermore, it has been shown that the surgeon and hospital volume strongly influence patient outcome [15]. Thus, all operations were performed electively by two experienced and specialized high-volume pediatric surgeons or under the supervision of these surgeons.

In conclusion, we have demonstrated that PHH develops more acutely with lower gestational age in very premature infants. The vast majority of these infants require conversion from temporary ventriculostomy to permanent VPS. However, in a subset of infants, progressive PHH develops later, and primary VPS insertion can be considered.

## Footnotes

1 Traditionally, subcutaneous CSF_Rs are named after the neurosurgeon Ayub K. Ommaya (1930–2008) and the pediatric surgeon Peter Paul Rickham (1917–2003).
